# Exploring Dense Breast Density in Mammography: A Comparative Analysis of Breast Cancer Risk

**DOI:** 10.7759/cureus.74026

**Published:** 2024-11-19

**Authors:** Jose D Cardona Ortegón, Sergio Valencia, Laura Campaña Perilla, Julián D Guerra Barón, Javier A Romero

**Affiliations:** 1 Radiology, University Hospital Fundación Santa Fe de Bogotá, Bogotá, COL; 2 Radiology, El Bosque University, Bogotá, COL; 3 Radiology, University of La Sabana, Chia, COL

**Keywords:** biopsy, bi-rads, breast density, dense breasts, malignancy, mammography, screening

## Abstract

Background: Breast density is a strong predictor of breast cancer. However, the difference in risk between breast density categories C and D remains inadequately explored. Given the low occurrence of extremely dense breasts, this investigation is crucial because it may lead to modifications in screening techniques for those with these conditions.

Objective: The objective of the study is to evaluate the difference in breast cancer risk among women undergoing mammography and biopsy at a tertiary referral hospital in Colombia, based on American College of Radiology Breast Imaging Reporting and Data System (BI-RADS) results in categories of breast density C (heterogeneously dense) and D (extremely dense).

Methods: This retrospective cross-sectional study recorded variables from the mammographic BI-RADS scale, as well as histological and clinical variables from digital medical records. A stratified analysis of lesion malignancy/benignity was conducted according to density category by mammography and histological findings. The association between mammographic breast density subclassification of dense breasts and the occurrence or not of pathology-defined malignancy was sought.

Results: A total of 107 patients with breast density in categories C and D were included, with 88.7% having heterogeneously dense breasts. The frequency of breast cancer was 32%. Infiltrating ductal carcinoma was the most frequently diagnosed malignancy (N = 14). A higher BI-RADS category was correlated with breast density grade D and malignancy. A statistically significant association (p <0.045, RR 1.95, CI: 1.12-3.50) was found when comparing breast density (categories C and D) with the risk of malignancy. The positive predictive value (PPV) varied across different BI-RADS categories (BI-RADS 4A 25% vs. 0%; p-value 0.005; BI-RADS 4B 50% vs 10.5%; p-value 0.032).

Conclusion: Efforts and resources should be focused on patients with extremely dense breasts, emphasizing the importance of additional (individualized) screening. Breast density could change the PPV within each BI-RADS category. However, further studies are needed to define the risk associated with each breast density subcategory and within BI-RADS categories, as well as to assess the efficacy of additional screening in patients with extremely dense breasts.

## Introduction

Breast cancer is currently one of the most diagnosed cancers in the world, with an estimated 2.26 million cases registered in 2020. It ranks as a leading cause of death among women, standing as the first cause of cancer mortality [1]. According to the Center for Disease Control and Prevention (CDC), approximately 272,454 patients are diagnosed with breast cancer each year in the USA, resulting in around 42,211 deaths annually [2]. In Colombia, the incidence of breast cancer was approximately 101,893 in 2018, compared to 71,442 in 2012, indicating an increase of 42.6% in incidence over six years. In 2019, it was reported as the eighth leading cause of death in Colombia [3]. The World Health Organization reported that up to 30% of cancers today are preventable. This opens the door to the need for both prevention and screening to mitigate mortality from this disease, especially in developing countries. In 2002, 54% of deaths from breast cancer occurred in low and middle-income countries [4].

Diagnostic imaging stands as one of the pillars of breast cancer screening, involving the assessment of breast tissue for possible lesions in asymptomatic women, with subsequent follow-up for suspicious lesions if required. This breast tissue comprises adipose tissue and fibroglandular parenchyma [5], with the proportion of fibroglandular parenchyma being a determinant of breast density in diagnostic images [6]. Changes in breast density are relevant not only because they reduce the sensitivity of mammography for lesion detection (masking effect) but also because they represent an independent risk factor for the development of breast cancer, increasing the need for imaging in these patients or changing the screening algorithm [7]. 

The most widely used classification for breast lesions observed through mammography is the one proposed by the American College of Radiology Breast Imaging Reporting and Data System (BI-RADS) [8]. This classification has been used to standardize the interpretation of diagnostic images, increase consistency among readers, and standardize radiologic reporting of breast images. It is classified from 1 to 5, depending on the suspicion of malignancy found in the diagnostic image or corresponds to category 6 in case of a confirmed diagnosis of breast cancer. Currently, breast density imaging findings are evaluated in a qualitative/visual analogous manner using the fifth edition of BI-RADS; four categories are defined to classify breast density, A (almost completely fatty), B (sparse fibroglandular), C (heterogeneously dense), and D (extremely dense) [9,10]. Based on this, two large groups are classified, patients with non-dense breasts (A and B) and patients with dense breasts (C and D).

## Materials and methods

**Figure 1:**
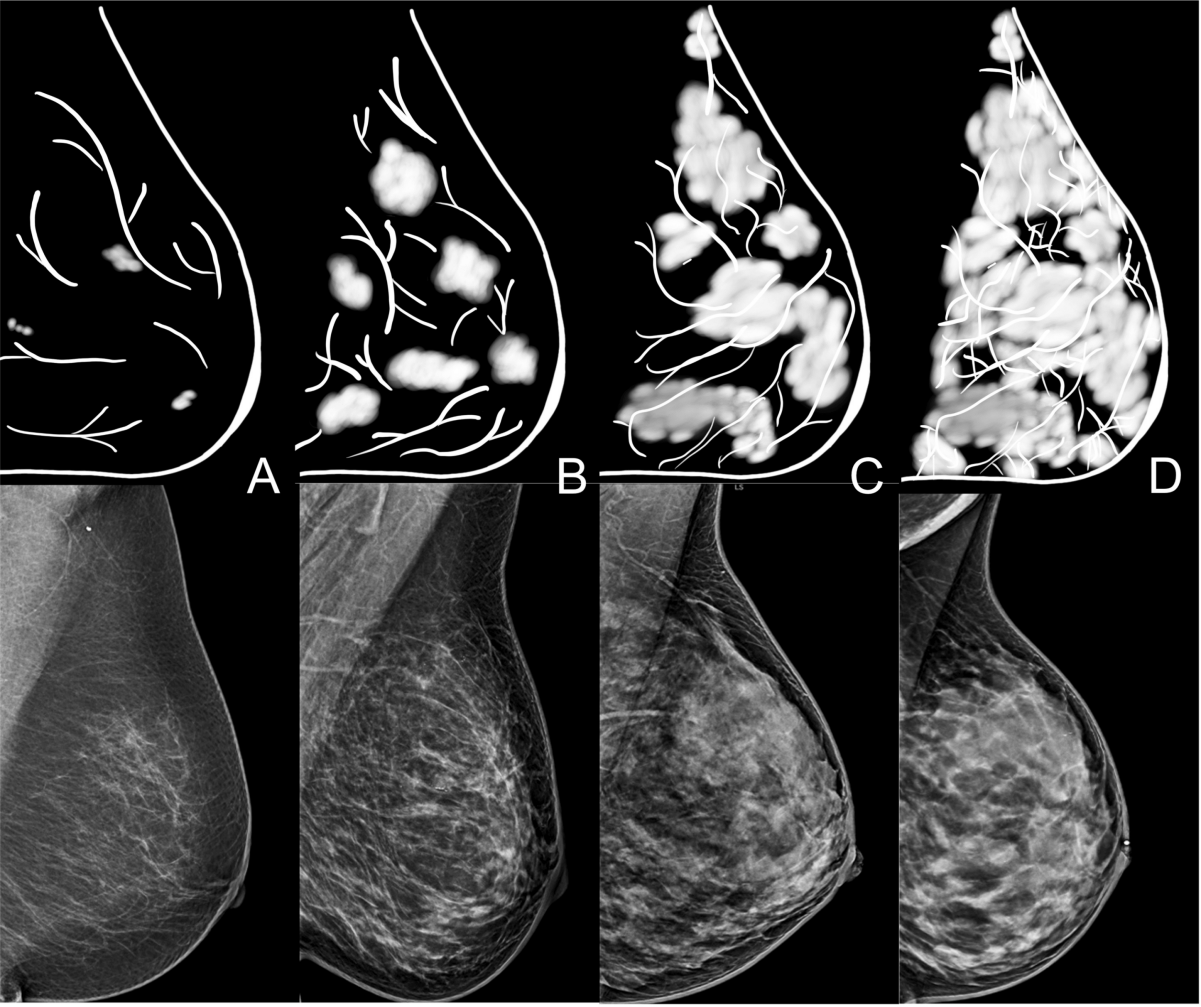
Mammography images and digital drawing illustrations showing the different categories of breast density. (A) Category A: almost completely fat; (B) category B: sparse fibroglandular; (C) category C: heterogeneously dense; and (D) category D: extremely dense.

This retrospective cross-sectional study aimed to identify the risk difference between sub-classifications of the mammographic breast density scale (Figure 1), specifically focusing on the levels of heterogeneously dense and extremely dense breasts (categories C and D) in women who underwent mammography and biopsy in a tertiary reference hospital (University Hospital Fundación Santa Fe de Bogotá) in Bogotá from January 2019 to December 2020.

Variables of BI-RADS 4A, 4B, 4C, 5, and 6 mammographic findings were recorded based on the structured reports in the PACS-IMPAX (Enterprise) system [11]. Additionally, histological and clinical variables, including cancer type, were collected from medical records. Demographic results, including age groups, and statistical tests were used to explore the association between the two subcategories of dense breasts (categories C and D) and the occurrence of malignancy as defined by pathology. Association statistics were reported as odds ratios (OR) with their respective 95% confidence intervals. A p-value less than 0.05 was considered statistically significant for all tests. The statistical analyses were performed using Jamovi 2022 software for Microsoft Windows version 2.3 [12,13].

The inclusion criteria for this study comprised patients who had undergone mammography within the institution and mammograms with BI-RADS categories 4, 5, and 6. Additionally, patients with biopsies and histopathological analyses performed at our institution that resulted in a diagnosis of malignancy were included.

On the other hand, exclusion criteria were applied to patients who had undergone biopsies outside the institution, patients with unavailable biopsy results, patients who had received institutional biopsies without institutional mammography, and patients with dense breasts and a diagnosis of benign neoplasia.

## Results

A total of 346 patients with mammography results with BI-RADS classifications 4A, 4B, 4C, 5, and 6 were included, with 164 (47%) having breast density in categories C and D. A total of 107 patients (65.2%) had a histopathologic evaluation (HPE) report following biopsy and were classified into BI-RADS categories amenable to biopsy (e.g., BI-RADS 4A, 4B, 4C, and 5). The remaining 34.8% did not have a histopathologic report or institutional follow-up. Most of the cases described involved patients analyzed between 2019 and 2020. Patients with heterogeneously dense (95 patients) and extremely dense (12 patients) breasts were selected, corresponding to breast density categories C (11.2%) and D (88.7%). The mean age of the population was 52.3 years, with a standard deviation of ± 11.5 years.

**Figure 2:**
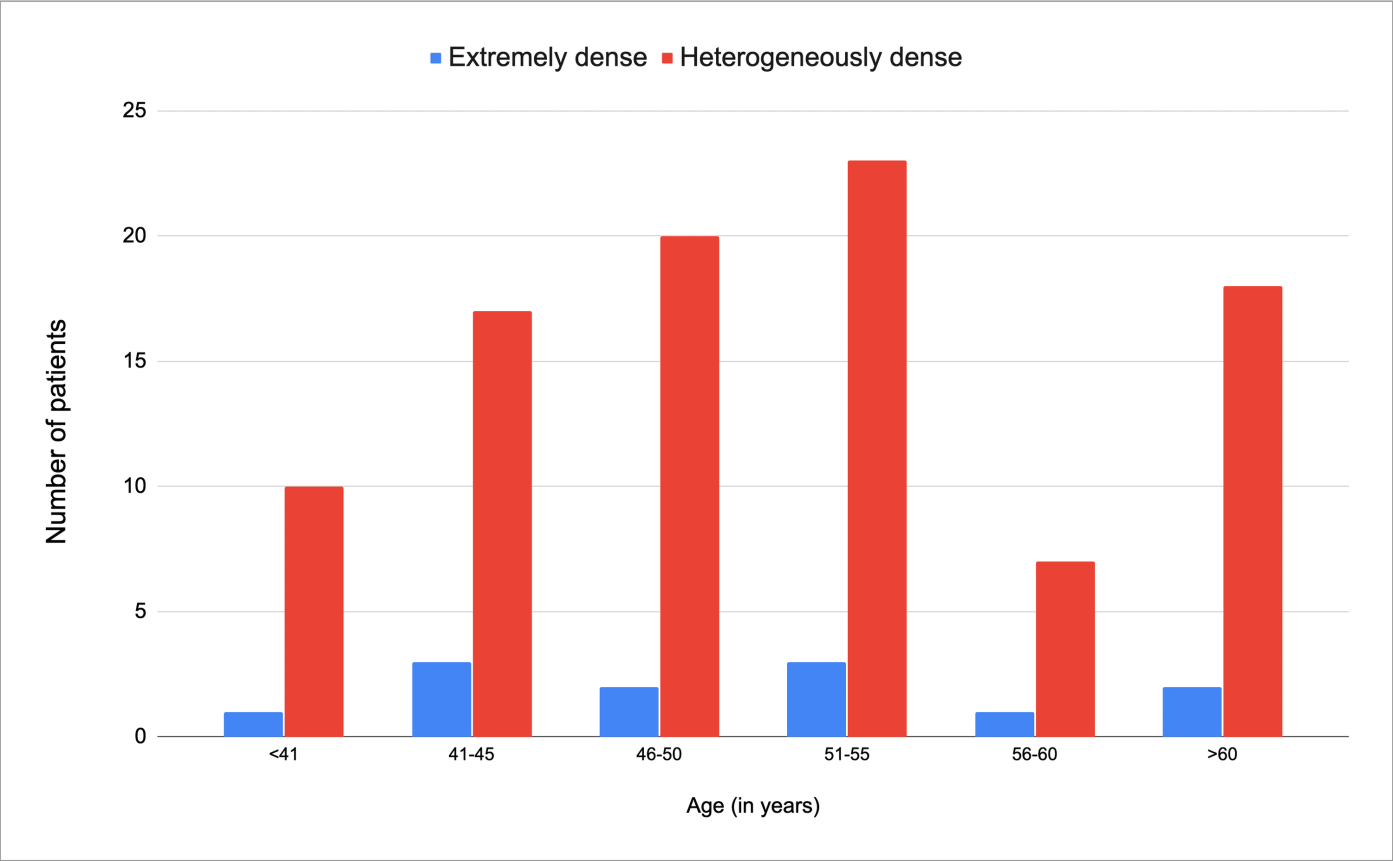
Histogram of the distribution of age groups according to breast density.

**Figure 3:**
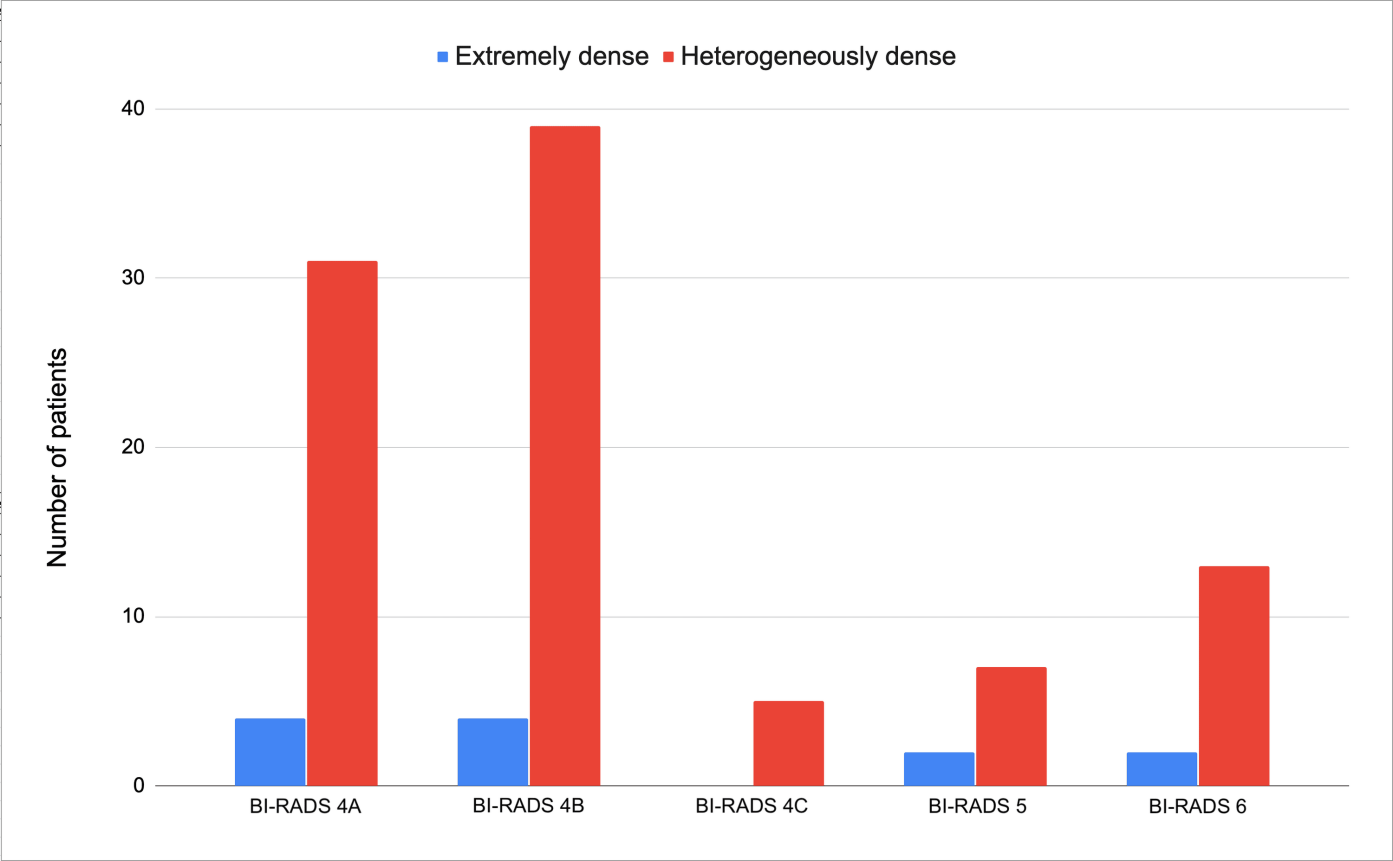
Histogram of breast density distribution according to BI-RADS categories.

Six age categories were created from the study group, patients younger than 41, 41 to 45, 46 to 50, 51 to 55, 56 to 60, and older than 60 years. No discernible trend was observed in the distribution of cases of patients with grade D density concerning age subgroups (Figure 2). Regarding breast density and BI-RADS classification, a greater number of heterogeneously dense cases were found among BI-RADS 4A and 4B cases, with no evidence of a greater distribution in type D density cases (Figure 3).

Ultrasound-guided percutaneous biopsy was used for tissue sampling in this study. The breast cancer frequency in the studied population was 32%. Fibroadenoma was the most prevalent benign histologic finding in the sample, with 35 cases (37%), while infiltrating ductal carcinoma was the most prevalent malignant finding, accounting for 14 cases (13.1%). The distribution of histological results and the BI-RADS classification revealed a clear trend in the BI-RADS category; the number of patients with breast density grade D also increased.

Statistical analysis showed an increase in the number of malignancy cases with higher BI-RADS classification categories, with a higher number of benign cases when the BI-RADS classification was lower. These results showed a statistically significant difference (p-value <0.001). Additionally, a statistically significant association was found when comparing breast density and histopathological results, showing a higher risk of malignancy in patients with extremely dense breasts. This result was statistically significant with a p<0.045 and a relative risk of 1.95 (CI: 1.12-3.50) (Table 1). Finally, a difference was observed in the positive predictive value (PPV) (percentage of positivity) for each BI-RADS category according to breast density, finding higher PPV for BI-RADS 4A and 4B categories in patients with extremely dense breasts (BI-RADS 4A 25% vs. 0% p-value 0.005; BI-RADS 4B 50% vs. 10.5% p-value 0.032).

**Table 1:** Contingency table comparing breast density to the histologic outcome.

Breast density	Histological result					
	Malignant	Benign	Total	Fisher's test	Relative risk	95% Confidence interval
Extremely dense	7	5	12	0.045	1.98	1.12 - 3.50
Heterogeneously dense	28	67	95			
Total	35	72	107			

## Discussion

Breast density, mainly in categories C and D, is linked to an increased risk of breast cancer. Among 1000 women with breast density type A, 10 women will develop breast cancer, while out of every 1000 women with breast density type D, the number increases to 32 [14]. In our study, we observed an increased risk of malignancy in breast density subcategory D (extremely dense), demonstrating a statistically significant association compared to other dense breast density subcategories. Specifically, the analysis identified that patients with breast density grade D have 1.9 times greater risk of developing breast cancer. The meta-analysis by Bodewes et al., comprising nine studies, revealed that breast density classified as D carries a two-fold increased risk compared to category B. However, they did not analyze the difference in risk between categories D and C, which is an important analysis as it allows for an adjustment that could guide personalized screening. In our study population, we observed a two-fold risk difference between these two categories (C and D) and identified that the PPV within each BI-RADS category could vary according to breast density [15].

In the U.S. population, approximately 43.3% of women aged 40 to 74 years have heterogeneous or extremely dense breasts (i.e., categories C and D) [16]. Another multicenter study in Lebanon found that 9.7% of women had type A breast density (almost completely fatty), 37.5% had type B breast density (sparse fibroglandular), 45.9% had type C breast density (heterogeneously dense), and 7% had type D breast density (extremely dense) [17]. In our population sampling, we studied subcategories C and D, finding a similar prevalence with both American and Lebanese women, with 47%. A similar result in another Colombian study reported 43.9%, of which only 4.29% of breasts were extremely dense [18].

Furthermore, this study also suggests that the PPV within each BI-RADS category could change according to breast density (BI-RADS 4A: 25% vs. 0%, p-value 0.005; BI-RADS 4B: 50% vs. 10.5%, p-value 0.032). This finding is relevant for modifying or guiding the screening in an individualized manner. 

This is of utmost importance as it could help to direct resources and efforts to the population at higher risk, thus improving diagnosis and treatment for this pathology of high morbidity and mortality. One strategy could involve additional (personalized) screening of patients with breasts classified as extremely dense since they have twice the risk of breast cancer compared to heterogeneously dense breasts. In the systematic review by Melnikow et al., the need for additional screening in patients with dense breasts (categories C and D) was analyzed, finding limited evidence suggesting that there is an increased diagnosis of breast cancer with additional screening with ultrasound or MRI; however, there is also an increase in recalls in patients without breast cancer [19].

Therefore, considering our findings and the reviewed studies, it may be beneficial to implement additional (personalized) screening for patients at a higher risk of breast cancer, specifically those falling into subcategory D. By adopting this approach, the financial burden on the healthcare system would be minimized, as the prevalence of breast density subcategory D is typically less than 7% in most populations. Furthermore, the impact on recall rates would be reduced, as mentioned previously. However, it is crucial to conduct prospective studies to assess the effects of supplementary screening on outcomes and determine if there is any potential for overdiagnosis of the disease.

Our study also identified an association between malignant breast histopathologic outcome and upstaging of breast lesions with BI-RADS classification, as expected and reported in the literature [20].

Artificial intelligence (AI) and computer-aided detection can reduce variability in breast density classification, providing a more standardized and objective approach. AI tools analyze mammographic images, improving lesion detection accuracy in cases of dense breasts, where mammography sensitivity is lower. However, while AI may help accurately select breast density, studies show that variability among expert radiologists is low, and their ability to correctly classify breast density is high [21].

This article has some limitations, such as bias in the subjective categorization of breast density, limited sample in the group of women with extremely dense breast density (category D), and the fact that this study was conducted at a single institution. Therefore, it is important to interpret the results with caution to extrapolate the findings.

## Conclusions

Breast density by mammography is a useful tool for determining breast cancer risk. Patients with extremely dense breasts are at increased risk of developing breast cancer and, therefore, should receive specialized care and closer surveillance with additional screening. Likewise, our study suggests that breast density changes the PPV within each BI-RADS category. However, it is important to conduct studies with larger numbers of patients and in diverse populations to further define the risk of each breast density subcategory and the usefulness of additional imaging in patients with extremely dense breasts in terms of diagnostic accuracy and clinical outcomes. Although there are promising new AI technologies, they require further validation and clinical adaptation to assess their real impact on breast cancer diagnosis and their role in differentiating breast density. These technologies require additional validation and clinical adaptation to assess their real effectiveness in differentiating breast density.
